# Multistressor global change drivers reduce hatch and viability of Lingcod embryos, a benthic egg layer in the California Current System

**DOI:** 10.1038/s41598-022-25553-z

**Published:** 2022-12-20

**Authors:** Ellen Willis-Norton, Mark H. Carr, Elliott L. Hazen, Kristy J. Kroeker

**Affiliations:** 1 University of California Santa Cruz, 130 McAllister Way, Santa Cruz, CA 95060 USA; 2 Environmental Research Division, Southwest Fisheries Science Center, National Marine Fisheries Service, National Oceanic and Atmospheric Administration, Monterey, CA 93950 USA

**Keywords:** Climate change, Climate-change ecology, Ecophysiology

## Abstract

Early life history stages of marine fishes are often more susceptible to environmental stressors than adult stages. This vulnerability is likely exacerbated for species that lay benthic egg masses bound to substrate because the embryos cannot evade locally unfavorable environmental conditions. Lingcod (*Ophiodon elongatus*), a benthic egg layer, is an ecologically and economically significant predator in the highly-productive California Current System (CCS). We ran a flow-through mesocosm experiment that exposed Lingcod eggs collected from Monterey Bay, CA to conditions we expect to see in the central CCS by the year 2050 and 2100. Exposure to temperature, pH, and dissolved oxygen concentrations projected by the year 2050 halved the successful hatch of Lingcod embryos and significantly reduced the size of day-1 larvae. In the year 2100 treatment, viable hatch plummeted (3% of normal), larvae were undersized (83% of normal), yolk reserves were exhausted (38% of normal), and deformities were widespread (94% of individuals). This experiment is the first to expose marine benthic eggs to future temperature, pH, and dissolved oxygen conditions in concert. Lingcod are a potential indicator species for other benthic egg layers for which global change conditions may significantly diminish recruitment rates.

## Introduction

As global environmental change progresses, species are being exposed to novel environmental conditions, creating unprecedented risks^1–3^. Physiological intolerance to future conditions can affect survival, reproductive capacity, growth and development rates, the timing of ontogenetic transitions, and recruitment dynamics^2–6^. Our ability to manage for the resilience of species and the ecosystems and economies many of these species support requires understanding how species will respond to likely changes in their environment. For the many marine species with complex life histories, not only do physiological susceptibilities differ markedly among life stages, but so does the ability to move to avoid physiological stressful conditions^6–10,11^. Predicting the consequences of changing environmental conditions for individual organisms, and the populations and communities they constitute, requires knowledge of how environmental stressors impact the physiological rates and tolerance of each lifestage of a species.

The first lifestage for many fish is as developing fish embryos, which are passive recipients of their environment and do not possess fully developed regulatory processes^12–14^. The vulnerability of this susceptible life stage is exacerbated for benthic egg masses that are bound to geologic or biogenic substrates and cannot evade locally unfavorable environmental conditions. Most freshwater and brackish fishes lay benthic eggs (e.g. sturgeon and salmon)^15^. Marine fishes typically produce pelagic eggs because of saltwater’s buoyancy^16^. However, small demersal fishes are often benthic egg layers, including over half of the species in the ornamental fish trade (e.g. blennies, gobies, damselfish)^17^. Well known, commercially important marine benthic egg layers also include Lingcod (*Ophiodon elongatus*), Cabezon (*Scorpaenichthys marmoratus*), Herring (*Clupea pallasii*), market squid (*Doryteuthis opalescens*), and Capelin (*Mallotus villosus*).

Here, we conducted a flow-through mesocosm experiment that exposed Lingcod eggs to temperature, pH, and dissolved oxygen (DO) conditions projected for the central California Current System (CCS) by the year 2050 and 2100. Adult Lingcod are high trophic level, voracious predators in northeast Pacific that lay benthic egg masses in shallow reef ecosystems^18^. Many Lingcod populations are found in coastal upwelling zones within the CCS from 29° to 58°N north^19^. Nearly 20% of global fish catch occurs in upwelling regions, despite representing less than 1% of the global ocean^20^. Temperature, pH, and DO covary in upwelling zones, as upwelled waters are cooler and have lower pH and DO concentrations than the surface waters they replace^21,22^. As global change progresses, temperature in the central CCS is projected to increase while pH and DO concentrations of upwelled waters are expected to continue to decrease due to ocean acidification and deoxygenation^23,24^. Lingcod’s ecological and economic importance in a highly-productive ecosystem with already depressed pH and DO conditions makes this experiment an important case study for how a benthic egg layers’ metabolism, growth, survival, and larval quality could be impacted by global environmental change.

Temperature is the most well-studied environmental variable and is often considered the dominant stressor that has a clear impact on developing embryos. Previous experiments exposing pelagic eggs and larval stage fish to temperature increases found that development time tends to decrease, metabolic rate increases, and hatch success decreases^12,25–28^. Additionally, larval quality (e.g. length, yolk-sac volume) is affected by the temperature eggs are exposed to, with evidence for decreased fitness^27,29–33^. Fish eggs can have higher DO requirements than adults and cannot avoid low oxygen environments^34^. In contrast to warming, reduced DO levels can slow development and metabolic rates, as well as reduce embryo survival, decrease size at hatch, and increase morphological abnormalities^35–38^. The story for ocean acidification is more complicated. Previous studies have found contradicting results regarding the effect of decreasing pH on fish eggs. Some studies have reported detrimental effects, such as reduced survival and larval quality^39–41^; whereas others have failed to find any effect^42,43^; and many studies show minor effects^44–47^. Understanding how the combined effects of warming, acidification and deoxygenation will affect fish eggs and embryos remains a critical next step for global change studies as it has been established that larvae born in poor condition have a low chance to survive and recruit into the adult population^48^.

While these previous studies examined the physiological response to individual stressors, global change will cause simultaneous shifts in all three, potentially interacting, stressors in the CCS and globally. Experimental studies examining the impact of changing temperature, pH, and DO (known as the “deadly trio”) are scarce but are urgently needed to provide more accurate predictions of biological responses to environmental change^49^. This study is the first to expose marine benthic eggs to future temperature, pH, and DO conditions in concert.

Predictions for how benthic eggs will be impacted by future conditions are difficult because of potential synergies between temperature, pH, and DO^50^. The addition of acidification and hypoxia as stressors could enhance sensitivity to the temperature signal^30,51^. Or the three stressors operating together could cause effects contrary to what might be predicted^52^. Lingcod embryos may already be physiologically stressed and even small decreases in pH and DO could be detrimental to development, or as benthic spawners that evolved in upwelling zones, Lingcod eggs may be adapted to cope with low DO and pH conditions^39,50^. To assess the biological ramifications of these global change conditions, experiments can take a full-factorial approach, providing a mechanistic understanding of all possible stressor interactions; or experiments can take a scenario-based approach, where multistressor treatments are based on projected future conditions^53^. We chose to employ a scenario-based approach because it permits robust replication and allows for hypotheses about the combined impact of the “deadly trio” that are relevant to managers and policy makers.

Since Lingcod are likely adapted to existing pulses of low pH and DO conditions associated with coastal upwelling, temperature may be a more dominant stressor impacting the viability of Lingcod eggs in 2050 and 2100. Based on results of the studies cited above, we hypothesize that conditions expected in Monterey Bay by the year 2050 and 2100 will (1) increase metabolic rate; (2) decrease time to hatch and reduce percent viable hatch; and (3) decrease capacity for growth and diminish larval quality. If metabolism, successful hatch, and larval quality is unaffected by the predicted environmental conditions evaluated in this experiment, it suggests that Lingcod may currently have the genetic and physiological scope to tolerate global environmental change. If the earliest life history stage is unable to cope with predicted future conditions, then the persistence of this benthic egg laying species in a changing ocean is uncertain.

## Results

### Metabolism of Lingcod embryos

We did not detect differences in the respiration rate of Lingcod embryos among the current, year 2050, and year 2100 environmental treatments (Supplementary Fig. S2). The comparable oxygen consumption rates between the year 2020 and year 2100 treatment indicates that 10-days before hatch, the embryos in the year 2100 treatment had not yet undergone a mass mortality event (see below). It is important to note that we assumed the 1 g egg masses in the respiration chambers were uniformly fertilized, which may have affected the likelihood of detecting differences in the embryos’ metabolic rates.

### Hatch timing and percent hatch

Degree days to first hatch and degree days to 50% hatch increased as temperature rose and pH and DO levels decreased across treatments, suggesting environmental change conditions retarded Lingcod embryo development (Table 1). Total hatch day significantly decreased from 4 days in the year 2020 treatment, to 3 days in the year 2050 treatment, to less than 1 days in the year 2100 treatment (Table 1).

**Table 1:** Mean and standard deviation of degree days and calendar days to first hatch, degree and calendar days to 50% hatch, and total number of hatch days for the year 2020, year 2050, and year 2100 treatments.

	Degree days to 1st hatch	Calendar days to 1st hatch	Degree days to 50% hatch	Calendar days to 50% hatch	Total number of hatch days
2020	256 ± 10	21 ± 0.8	263 ± 7	21 ± 0.6	4 ± 1
2050	281 ± 13	20 ± 0.9	287 ± 14	21 ± 0.9	3 ± 1.4
2100	320 ± 13	21 ± 0.8	320 + 13	21 ± 0.8	0.3 ± 0.6
p < 0.01	Yes	No	Yes	No	Yes

GLMMs with a Poisson distribution were run to determine statistical significance. Pairwise comparisons for degree days to first hatch (p_2020,2050_ = 9 × 10^−7^, p_2050,2100_ = 1 × 10^−6^, p_2020,2100_ = 0), degree days to 50% hatch (p_2020,2050_ = 4 × 10^−6^, p_2050,2100_ = 8 × 10^−5^, p_2020,2100_  = 0), and total number of hatch days (p_2020,2050_ = 0.09, p_2050,2100_ = 7 × 10^−8^, p_2020,2100_ = 0) were significantly different. Calendar days to first hatch (p_2020,2050_ = 0.92, p_2050,2100_ = 0.86, p_2020,2100_ = 0.96) and calendar days to 50% hatch (p_2020,2050_ = 0.86, p_2050,2100_ = 0.95, p_2020,2100_ = 1) were not significantly different.

**Figure 1:**
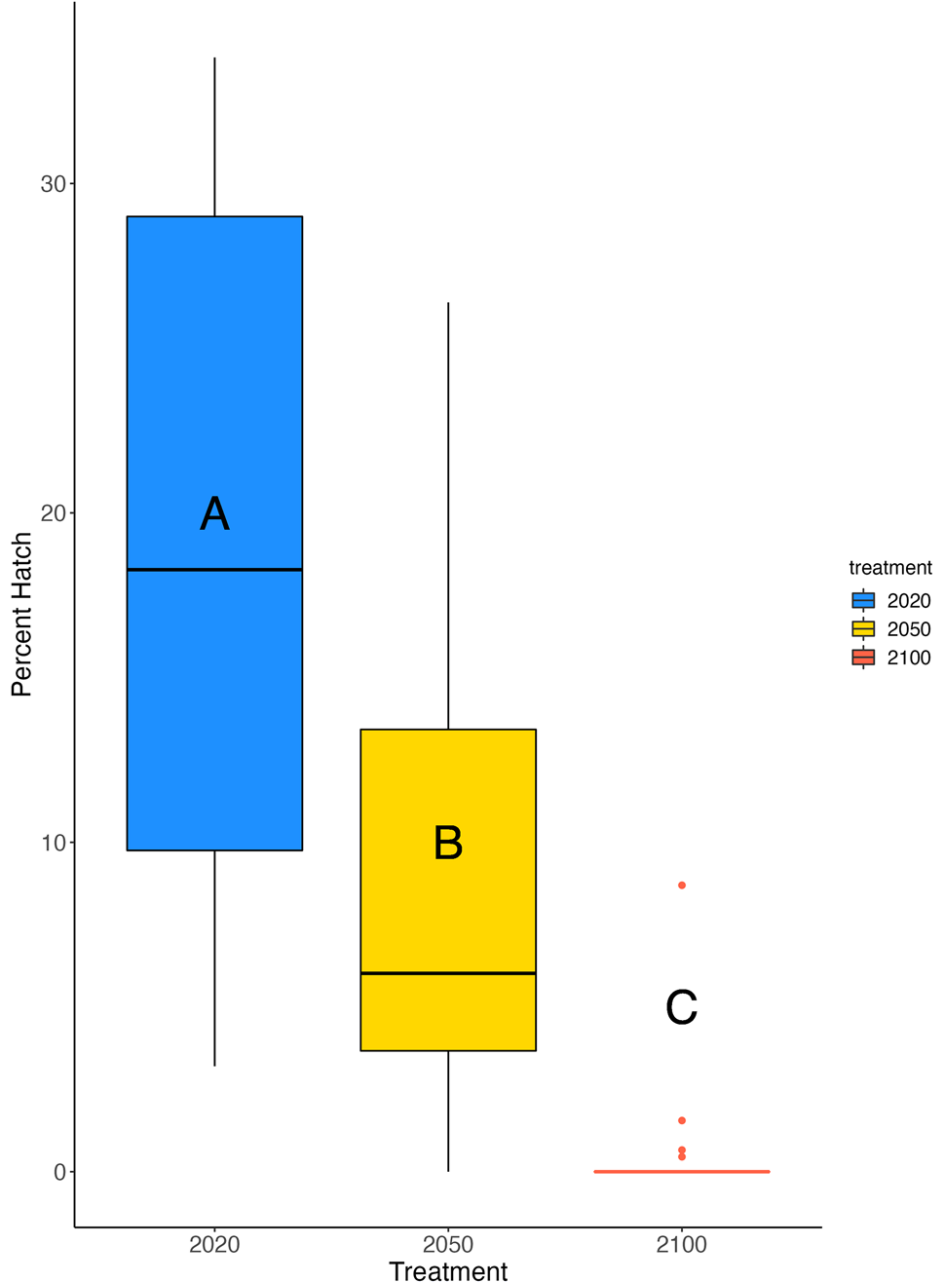
Boxplots illustrating the median, upper and lower quartile, and interquartile range of percent total hatch for the year 2020 (blue), year 2050 (yellow), and year 2100 (red) treatments. The average percent hatch was 19% for the year 2020 treatment, 9% for the year 2050 treatment, and 0.6% for the year 2100 treatment. GLMMs with a beta distribution were run to determine statistical significance, letters indicate significant differences (p_2020,2050_  = 6 × 10^–4^, p_2050,2100_ = 6 × 10^–6^, p_2020,2100_ = 7 × 10^13^).

Percent hatch ranged from 33 to 0% across the three treatments. Percent hatch was reduced for embryos experiencing environmental change conditions; the average percent hatch was 19% for the year 2020 treatment, 9% for the year 2050 treatment, and 0.6% for the year 2100 treatment (Fig. 1). The number of larvae that hatched with intact oil globules, signifying higher lipid reserves, was half of the total hatch for all three treatments (Supplementary Fig. S3). Similarly, once deformities that reduce swimming and feeding capability were cataloged, the number of larvae that hatched without any deformities reduced hatch success to 7% for the year 2020 and 3% for the year 2050 (Supplementary Fig. S3). All year 2100 larvae possessed deformities.

### Larval quality

**Figure 2:**
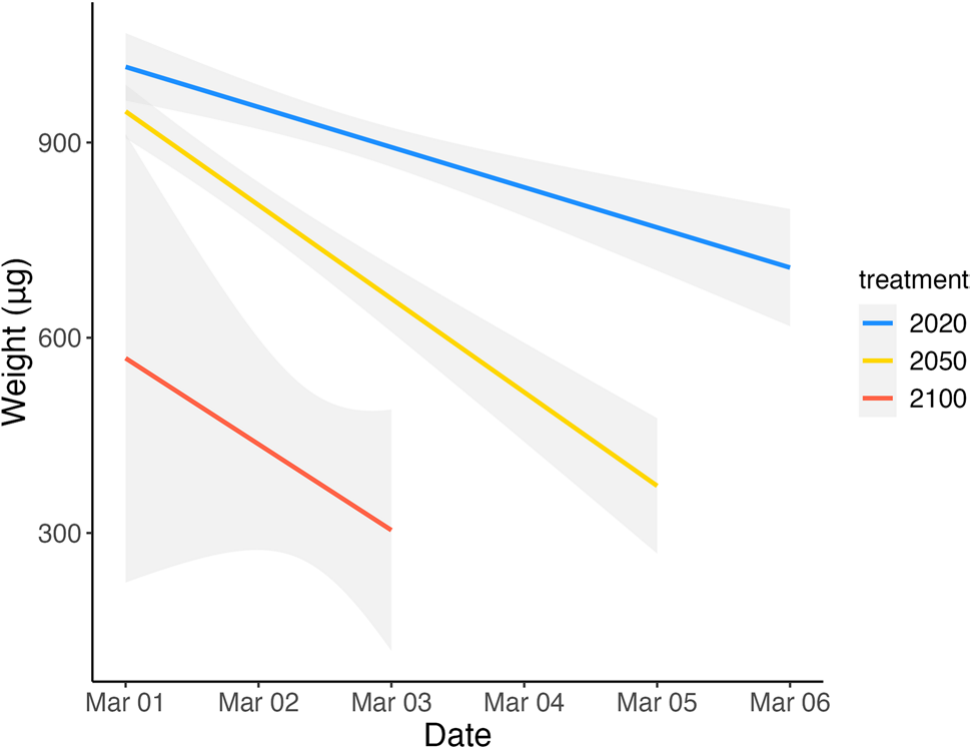
Linear regression for Lingcod larvae weight (μg) as the hatch period progresses for the year 2020 (blue), year 2050 (yellow), and year 2100 (red) treatment. A nested mixed model ANOVA was run to determine statistical significance (p_2020,2050_ = 0.21, p_2050,2100_ < 1 × 10^–4^, p_2020,2100_ < 1 × 10^–4^).

Larval quality was impacted by environmental change conditions. Larval weight was significantly lower in the year 2050 and year 2100 treatments compared to the year 2020 treatment (Fig. 2). Weight decreased as hatch progressed, likely because eggs on the interior of the 1 g egg masses hatched later and received less oxygen throughout development^54^. The total length and body depth of the Lingcod larvae declined as temperature increased and pH and DO decreased; although the year 2050 and year 2100 treatments were not statistically different (Table 2). Fulton’s body condition factor (K = weight/length^3^) was not significantly different between treatments, although the year 2100 treatment had an average K that was lower than the year 2020 and year 2050 average (Supplementary Fig. S4). Yolk area was unaffected by year 2050 conditions (1.54 and 1.58 mm^2^ in year 2020 and year 2050 conditions respectively), but yolk area dropped to an average of 0.58 mm^2^ in the year 2100 treatment. We did not detect differences among treatments for head length, head depth, snout length, eye diameter, and oil globule area (for larvae that had oil globules).

**Table 2:** Mean and standard deviations of morphometric data for Lingcod larvae samples from year 2020, year 2050, and year 2100 treatments.

	2020 (n = 295)	2050 (n = 282)	2100 (n = 17)
Total length	7.34 ± 0.77 mm^a^	6.97 ± 0.97 mm^b^	6.10 ± 1.16 mm^b^
Preanal length	3.76 ± 0.36 mm^a^	3.63 ± 0.55 mm^b^	3.23 ± 1.17 mm^b^
Postanal length	3.65 ± 0.54 mm^a^	3.53 ± 0.72 mm^ab^	3.01 ± 0.74 mm^b^
Head length	4.87 ± 2.94 mm^a^	4.00 ± 2.90 mm^a^	1.62 ± 0.59 mm^a^
Snout length	0.28 ± 0.34 mm^a^	0.29 ± 0.08 mm^a^	0.25 ± 0.09 mm^a^
Head depth	1.50 ± 4.35 mm^a^	1.24 ± 0.16 mm^a^	1.14 ± 0.17 mm^a^
Body depth	0.79 ± 0.11 mm^a^	0.71 ± 0.12 mm^b^	0.67 ± 0.14 mm^b^
Eye diameter	0.67 ± 0.06 mm^a^	0.64 ± 0.08 mm^b^	0.58 ± 0.12 mm^c^
Yolk area	1.54 ± 0.32mm^2a^	1.58 ± 0.35mm^2a^	0.59 ± 0.81mm^2b^
Oil globule area	0.11 ± 0.30mm^2a^	0.07 ± 0.08mm^2a^	0.09 ± 0.05mm^2a^

Count (n =) represents the number of larvae measured using ImageJ for each treatment, not the total hatch number. Treatments with different letters for each response variable were significantly different (nested mixed model ANOVAs; p < 0.01).

Occurrences of spinal, anal/gut, jaw, head, eye, and c-shaped deformities were catalogued for each treatment (Table 3). For every type of deformity, the larvae from the year 2020 treatment had the lowest instances of deformities, followed by the year 2050 treatment (although the two treatments were statistically equivalent); the year 2100 treatment had the highest instances of deformities and was significantly different from the other two treatments in all deformity categories except for the number of spinal deformities and c-shaped larvae.

**Table 3:** Mean and standard deviation for the percent of larvae from each 1 g Lingcod egg mass that had a deformity in the seven deformity categories for the year 2020, year 2050, and year 2100 treatments.

Deformity category	Example	% with deformity		
		2020	2050	2100
C-shaped		4.1% ± 2%^a^	7.8% ± 3.2%^a^	11.1% ± 13.7%^a^
Spinal deformity		15.5% ± 7.6%^a^	17.5% ± 10.5%^a^	44.4% ± 39.4%^a^
Anal/gut deformity		2.2% ± 1.7%^a^	8.3% ± 4%^a^	58.3% ± 38%^b^
Jaw deformity		8.2% ± 5%^a^	18.8% ± 8.6%^a^	88.9% ± 19.2%^b^
Malformed head		4.2% ± 3.8%^a^	11.7% ± 5.4%^a^	72.2% ± 29.3%^b^
Eye deformity		2.6% ± 2.2%^a^	6.6% ± 3.3%^a^	50% ± 43.3%^b^

N represents the number of images analyzed for each treatment. GLMMs with a beta distribution were run to determine statistical significance, letters indicate significant differences (p < 0.01).

Spinal deformities were the most common deformity seen in the year 2020 treatment (15.5% of larvae) and spinal and jaw deformities were the most common for the year 2050 treatment (17.5% and 18.8% respectively). Between 44 and 89% of larvae in the year 2100 treatment had a spinal, anal/gut, jaw, head, or eye deformities.

**Figure 3:**
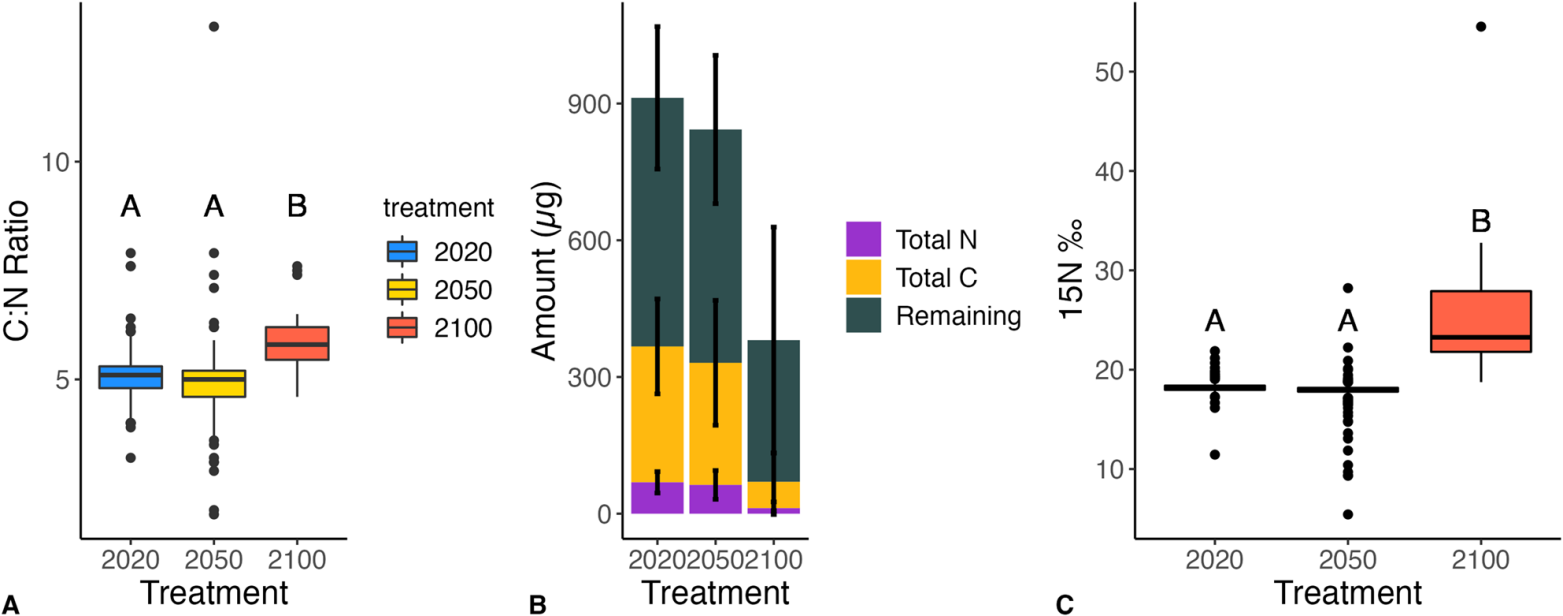
(**A**) Boxplots illustrating the median, upper and lower quartile, and interquartile range of the C:N ratio for the year 2020 (blue), year 2050 (yellow), and year 2100 (red) treatments (p_2020,2050_ = 0.27, p_2050,2100_ < 1 × 10^–4^, p_2020,2100_ < 1 × 10^–4^). (**B**) fraction of total weight (μg) comprised of total N and total C for each treatment. C) Boxplots illustrating the median, upper and lower quartile, and interquartile range of the concentration (per mille) of nitrogen-15 in each treatment (p_2020,2050_ = 0.57, p_2050,2100_ < 1 × 10^–4^, p_2020,2100_ < 1 × 10^–4^). Nested mixed model ANOVAs were run to determine statistical significance, letters indicate significant differences.

The C:N ratios for the 2020 and 2050 treatment were equivalent, while the C:N ratio was significantly higher for the year 2100 treatment (Fig. 3A). Lipid content remains relatively stable throughout fish embryo development, while protein content reserves are depleted, indicating differences in C:N ratios were due to changes in protein content^55^. The weight and the total nitrogen content (amount of protein) of the year 2100 larvae were substantially lower than the other two treatments (Fig. 3B). The year 2100 treatment also had a significantly higher concentration of nitrogen-15 (Fig. 3C). The year 2020 and year 2050 larvae had the same concentration of nitrogen-15, indicating the larvae in the year 2050 treatment were smaller, but maintained their protein reserves (supported by the two treatments’ similar average yolk areas).

## Discussion

Our results suggest that the combined effects of multiple environmental change stressors could reduce future recruitment rates of Lingcod in a coastal upwelling system. Projected temperature, pH, and DO concentrations by the year 2050 halved the successful hatch of Lingcod embryos. Larvae reared in the year 2050 conditions were viable (abnormalities were statistically similar to normal larvae) and had sufficient protein reserves, but were significantly smaller. Even slight changes in growth can impact recruitment, as smaller size leads to reduced swimming speeds, lower encounter rates with food, and higher risk of predation^36,43,48,56,57^. In the absence of acclimation or adaptation, our findings suggest egg and larval survival could plummet by the year 2100. In 2100 conditions, hatch was minimal (only 0.6% ± 2% of eggs hatched); larvae were undersized; yolk reserves were exhausted; larvae were concentrating nitrogen-15, suggesting that day-1 larvae were already metabolizing their protein reserves^58,59^. and deformities that reduce the ability to feed were widespread. Larvae that do hatch in these conditions will require abundant prey resources immediately after hatch occurs^31^. The likelihood of meeting those favorable conditions is low because the total number of hatch days declined significantly with increasing temperature and decreasing pH and DO. A longer hatch period increases the likelihood that at least a portion of day-1 larvae would encounter an upwelling event, which produces favorable conditions for growth^60^.

Our results are consistent with previous studies that found that in the northern CCS, temperatures above local natural variability reduced Lingcod larval survival and size while morphological abnormalities accumulated^29,61^. In fact, we obtained similar results even with a temperature difference between treatments that was half that of Cook et al.^30^, indicating synergies between the three stressors could have led to our observed effects. Another early Lingcod study examined egg masses at field sites in the San Juan Islands, Washington, and found that at 85% DO saturation, mortality was 6% while at 50% DO saturation mortality increased to 48%^54^. DO saturations from our 2050 and 2100 treatments (80% and 70%, respectively) suggest that low DO conditions likely contributed to Lingcod embryo mortality. The majority of Lingcod eggs will be exposed to even lower DO saturations since the interior of egg masses has low interstitial oxygen levels compared to the periphery. The high mortality in the year 2100 treatment happened after our metabolism trials concluded, 9 days before hatch. Oxygen requirements increase as fish embryos grow larger in their egg case^36,62^. It is possible that embryos in the year 2100 treatment could tolerate the low DO conditions until their oxygen requirements increased in the final stages of development. For the larvae that did survive until hatch, the morphological abnormalities we observed in the year 2100 treatment were similar to those found in previous studies manipulating temperature, pH, and DO concentrations independently^29,35,59^.

We anticipated Lingcod embryos would develop faster in the future scenario treatments, since previous studies examining the impact of temperature on the egg stage of CCS groundfish found that time to hatch was faster at higher temperatures^29,61,63^. In this study, however, degree days to hatch was longer in the year 2050 and year 2100 treatments compared to the year 2020 treatment. This result could also be due to low DO; Giorgi (1984) found hatching of Lingcod embryos was protracted for poorly ventilated eggs. Ocean deoxygenation has received less attention in the literature, but it is one of the most drastic impacts expected with climate change^49^. In fact, a meta-analysis of experimental research on climate change stressors found that on average hypoxia has a stronger negative impact on marine fish performance than ocean warming^49^. If low DO slowed Lingcod embryo development, future DO conditions may decrease growth rates of benthic egg laying species, resulting in poorer foraging ability and increased predation risk.

Our hypothesis that metabolic rate would increase in future environmental change conditions was also not supported. Increasing or decreasing metabolic rate in response to stressors is often adaptive, improving the likelihood of embryo survival^30^. We predicted metabolic rate to rise because of the strong positive relationship between temperature and increased oxygen consumption^12,28^. However, both ocean acidification and hypoxia can cause metabolic depression^30,38,39,51,59^. Dahlke et al.^15^ found that at extreme temperatures, an interactive effect between low pH and high temperature caused a reduction in oxygen consumption by Atlantic cod (*Gadus morhua*) embryos. It is also possible that Lingcod embryos’ metabolic rate simply cannot adjust to stressful conditions, explaining why we did not see a difference in oxygen consumption between treatments.

Our results demonstrate that single stressor experiments likely do not capture the true impact of global environmental change on marine organisms. Experimental studies need to move toward multistressor scenarios that include temperature, pH, and DO to obtain more accurate predictions of biological responses to global change^49^. This is especially important in coastal upwelling systems that fuel the productivity of fish populations around the world. Lingcod are a potential indicator species for other benthic egg layers, including those adapted to relatively low pH and DO conditions. Our study makes evident that global change conditions can diminish marine benthic egg condition and depress recruitment rates. The size and persistence of Lingcod populations in the highly productive central CCS is dependent on acclimation or adaptation to these future conditions.

Parental acclimation to environmental change conditions could increase embryos’ physiological tolerance of stressful conditions. Many studies have found that acclimating mothers and fathers to conditions the offspring are exposed to helps mediate negative impacts in the embryos^12,64,65^. Other studies have found parental exposure does not improve tolerance; maternal exposure to stressful conditions may even reduce egg size and inhibit reproduction^12,66^. Future experiments should attempt to capture adult benthic egg-layers and induce spawning in captivity to incorporate the effects of parental exposure to multistressor global change scenarios. These experiments could also determine if Lingcod reproduction is inhibited in physiologically stressful conditions. Although field experiments have shown that Lingcod egg masses are found in hypoxic environments, thermal inhibition of reproduction is common across marine fish taxa^12^.

Despite our experimental finding of Lingcod egg’s high sensitivity to environmental change conditions, there is considerable gene flow throughout their range, suggesting increased genetic diversity could support adaptation^67^. For example, Lingcod are adapted to their local thermal environment. Cook et al.^30^ examined the effect of temperature on Lingcod embryos taken from the Seattle Aquarium and found that hatch was highest at 9 °C and no hatch occurred at 15 °C; the average temperature in our year 2020 treatment was 13 °C and hatch occurred in the 15.7 °C treatment. Lingcod age at maturity is between two to four years, indicating there may be enough generations between today and 2050 to adapt to future temperature, pH, and DO conditions^68^. Lingcod can live up to 25 years so older mothers are less likely to be adapted to environmental change conditions. Older mothers produce more larvae that grow faster and are more resistant to starvation^69,70^. Even in the ideal scenario where all young mothers are adapted to future conditions, recruitment rates in the central CCS will likely still decline because viable hatch from the most productive mothers will decrease.

Species’ vulnerability to environmental change is based on their exposure, sensitivity, and adaptive capacity to future conditions. Our study is the first to experimentally demonstrate that a marine benthic egg layer in an upwelling ecosystem is highly vulnerable to environmental change conditions projected by the year 2050 in their environment. Adaptive and responsive fisheries management will be needed to maintain Lingcod’s role as an important generalist predator and as an economically important fisheries species. If the pelagic egg and larval stages of other fishes from upwelling systems are as sensitive to global environmental change as benthic egg layers, we will potentially face a re-shaping of one of the most highly productive marine ecosystems. Ultimately this study can serve as an important case for coastal fisheries of the Northeast Pacific, requiring climate-ready management approaches in the face of changing ocean conditions.

## Methods

Our study design and our scientific collection permit was approved by the California Department of Fish and Wildlife (CDFW). The department has this authority through section 650, Title 14, of the California Code of Regulations. All methods were carried out in accordance with CDFW guidelines and regulations. In addition, the methods described below are in in accordance with the ARRIVE guidelines.

### Study system and egg collection

#### Study species

Lingcod are distributed along the west coast of North America from Southeast Alaska to Baja California, Mexico and are caught in both commercial and recreational fisheries across its entire range^36,71^. In Monterey Bay, a diverse and productive ecosystem of the central California Current System (CCS), Lingcod is an ecologically significant predator that contributes to economically important recreational and commercial fisheries. Spawning occurs from November through early spring with females depositing eggs in shallow rocky reefs with swift flow to provide adequate ventilation for the benthic egg masses^54,71,72^. One Lingcod egg mass is sired by one mother and between one and five fathers^73^. Males guard and fan the mass from fertilization until hatch, approximately six to seven weeks in the central CCS, and hatching takes place over three to seven days^71^.

#### Study area and field sampling

Lingcod benthic egg masses were collected from rocky reefs along the northern coast of the Monterey Peninsula, at the southern end of Monterey Bay, central California (36°38.4′N, 121°56′W; Supplementary Fig. S1). Male Lingcod guarding and tending benthic egg masses are often associated with high relief, nearshore kelp forests during spawning season. To obtain egg masses, we searched for male Lingcod with SCUBA at depths of 5 to 15 m bottom depth starting in early January 2020. Once on the vessel, egg masses were placed in large coolers filled with aerated seawater, transported to the NOAA Fisheries Santa Cruz laboratory on the University of California’s Coastal Science Campus, and placed in a flow-through seawater system.

Once in the laboratory, the developmental stage of the eggs was evaluated. We required eggs at an early stage of development to ensure that exposure to future conditions could continue throughout their embryonic development. On February 7th, 2020 one ~ 8 kg egg mass was collected at a reef off of Point Pinos, Pacific Grove that was fertilized approximately 1.5 weeks prior to collection. The egg mass was likely derived from one female spawner and multiple fathers (previous field studies identified between 1 and 5 fathers per egg mass)^74^. Stable isotope analyses indicated that the mother’s diet included piscivorous fish, suggesting high maternal provisioning^75^. Similar to Cook et al.^30^, we assumed the egg mass was uniformly fertilized. The egg mass was placed in a well-aerated holding container for three days to acclimate to the laboratory environment before being exposed to the experimental treatments.

### Experimental design

**Figure 4:**
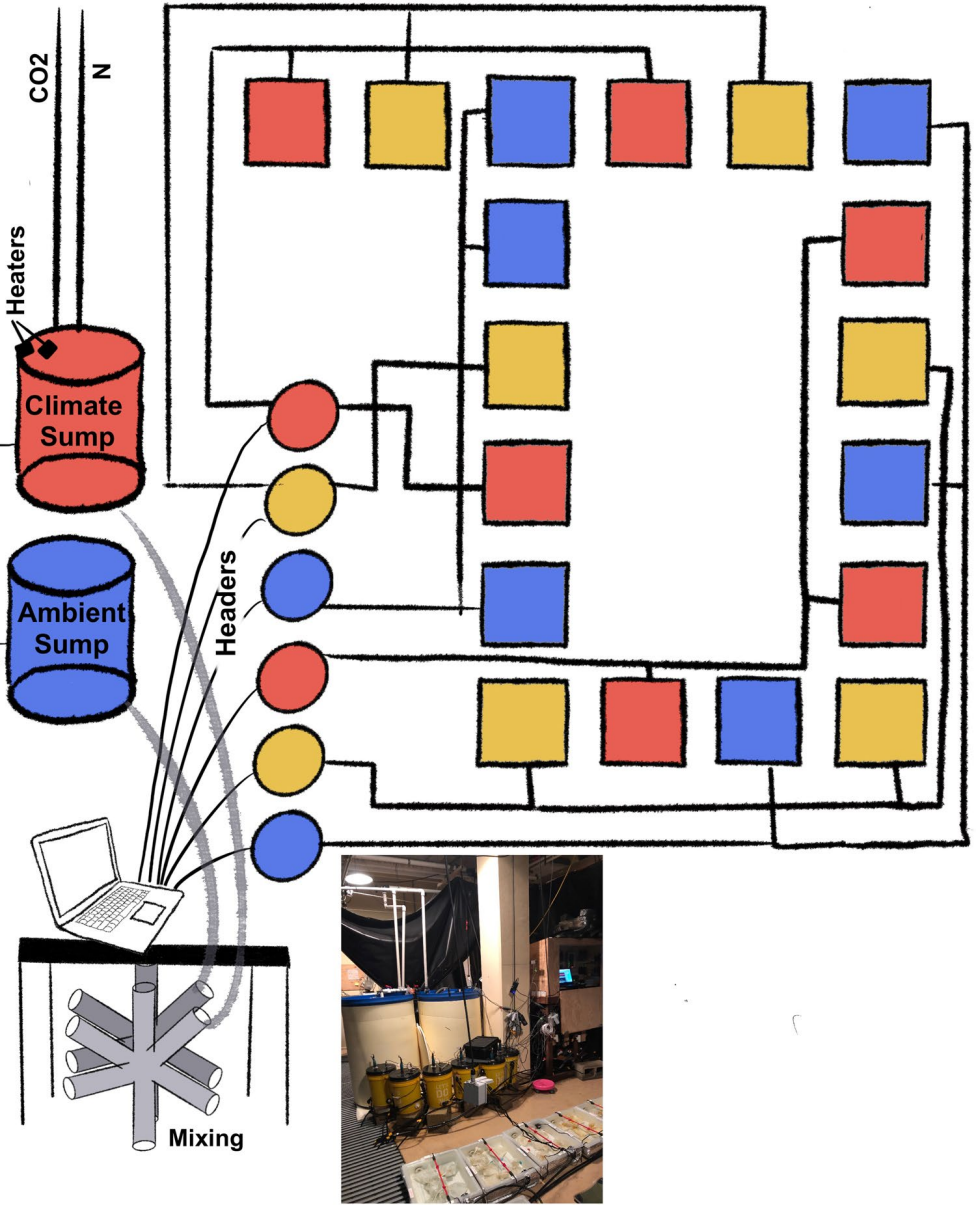
Conceptual figure illustrating the flow-through experimental mesocosm. Blue represents the year 2020 treatment, yellow represents year 2050, and red represents year 2100. The ambient and climate change sump tank water (cylinders) is mixed as it flows to the header tanks (circles) to attain the setpoints for each treatment. There are two replicate header tanks for each treatment and each header tank supplies water to three replicate aquaria (squares), for a total of six replicate aquaria for each treatment.

The flow-through mesocosm was designed to simultaneously manipulate temperature, pH, and DO levels of the incoming seawater to create three treatment levels: (1) year 2020 conditions, (2) projected year 2050 conditions, and (3) projected year 2100 conditions (Fig. 4). The setpoints for these three treatments were based on an ensemble of 25 climate change models assembled by the Coupled Model Intercomparison Project (CMIP5) run with RCP8.5 and nearshore ocean acidification projections for the CCS^23,24,76–78^. Based on the literature, we assumed that the average depth of Lingcod egg masses was 10 m, and adjusted the future temperature, pH, and DO setpoints accordingly (based on observations from Monterey Bay moorings^78^. The setpoints for the three treatments were:

1. Year 2020—Temperature: 13 °C; pH: 7.9; DO: 9 mg/l
2. Year 2050—Temperature: 14.5 °C; pH: 7.7; DO: 7 mg/l
3. Year 2100—Temperature: 15.8 °C; pH: 7.5; DO: 5 mg/l

The experimental system was fed with raw seawater pumped through a UV filter into two large sump tanks (~ 350 l). Sump tank 1 (“ambient sump”) had a chilled seawater line to maintain an average water temperature of 12.5 °C. pH and DO levels were unmanipulated. Sump tank 2 (“global change sump”) had four 1000-W heaters to maintain temperatures between 17 and 21 °C, the range mimicking the diel temperature cycle. The global change sump also had a CO_2_ gas line flowing at 0.5 sl/min to reduce pH levels to 7.2, and a N_2_ gas line bubbled at 3 sl/min to reduce DO levels to 5 mg/l.

To achieve the three treatment setpoints, controllers (Universal Dual Analyzer, Honeywell) connected to tris buffer-calibrated sensors (Durafet, Honeywell) monitored pH and temperature in six 20 l header barrels (two headers per treatment) that were receiving a mix of water from the ambient and global change sump tanks through a distribution manifold. If pH exceeded the desired setpoint for a header tank, the corresponding solenoid valve opened to release water from the global change sump tank; if pH fell below the setpoint the solenoid valve closed. Water from the header tanks gravity-fed three replicate aquaria each, resulting in a total of six 14 l replicate aquaria per treatment (n = 18). Sections of the collected egg mass were ultimately placed in these 18 replicate aquaria.

**Figure 5:**
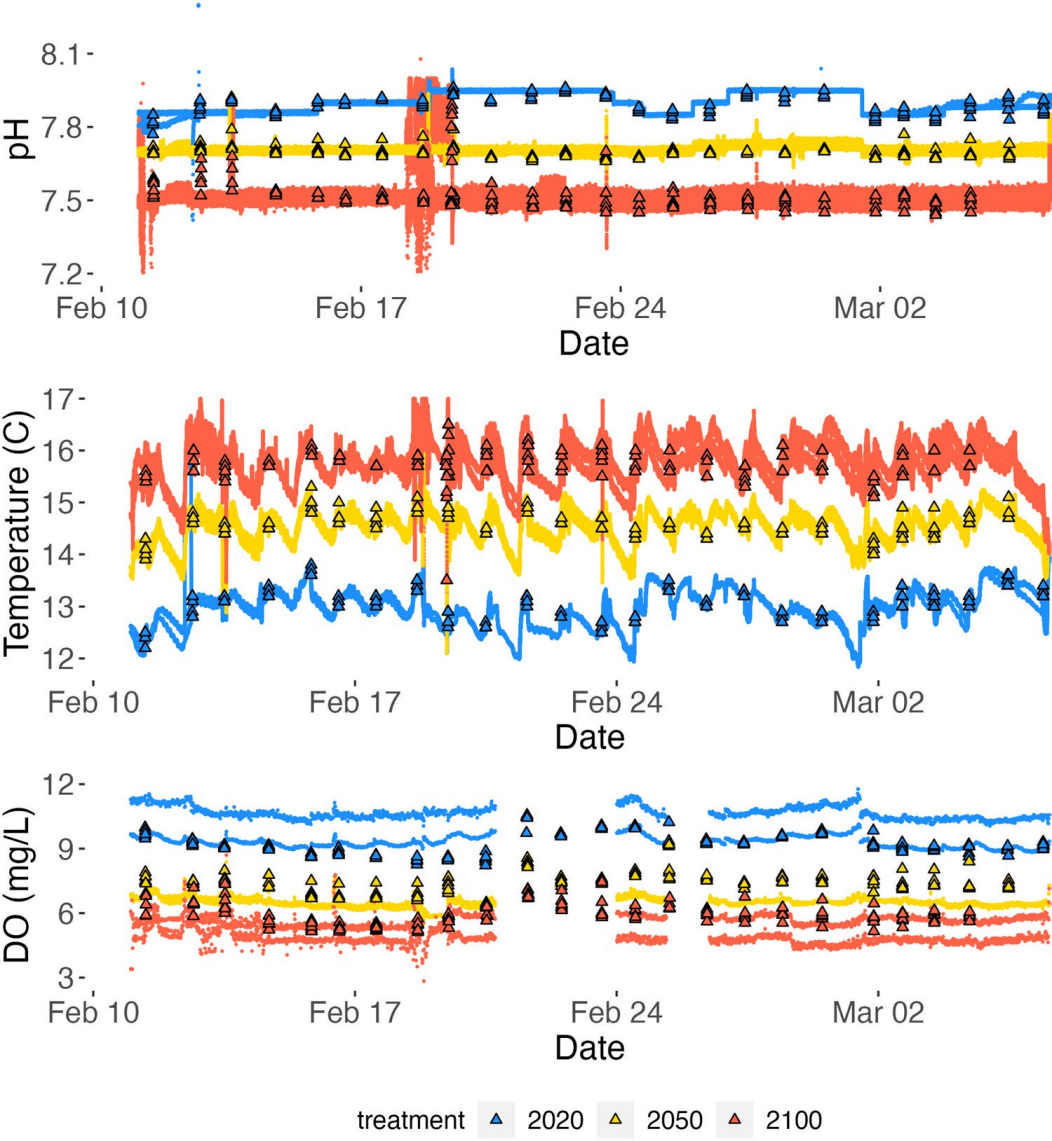
Temperature, pH, and DO values measured over the duration of the experiment for the year 2020 (blue), year 2050 (yellow), and year 2100 (red) treatments. Temperature and pH values were measured every 15 s by Durafet sensors in the header tanks (lines) and once daily by YSI sensors in the replicate aquaria (triangles). DO concentration was measured by Vernier sensors every 15 min in the header tanks (lines) and once daily by YSI sensors in the replicate aquaria (triangles).

Aquarium pumps in sump tanks, header barrels, and replicate aquaria ensured adequate mixing and water flow over the eggs; and timers controlled light levels to mimic the day-night cycle. Durafet sensors measured pH and temperature every 15 s and dissolved oxygen probes (GoDirect, Vernier) measured DO every 15 m in the sump tanks and header barrels. In the replicate aquaria, tris buffer-calibrated handheld sensors (YSI, Oakton Instruments) were used daily to measure pH, temperature, DO, and salinity. Discrete water samples were collected at three timepoints from the replicate aquaria to calculate additional carbonate chemistry parameters (Supplementary Table S1). Total alkalinity from these discrete samples was measured using a Metrohm 815 Robotic USB Sample Processor XL and Titrando 905. Throughout the month-long experiment, the average pH, temperature, and DO recorded in the headers and replicate aquaria were comparable to the three treatments’ setpoints (Fig. 5 and Table 4). At one point during the experiment (Feb. 18) a Durafet sensor for the year 2100 treatment was recording erroneous pH values, so the flow of water from the global change sump tank was controlled manually until the Durafet was replaced (Fig. 5).

**Table 4:** Mean and standard deviation of the temperature, pH, and DO conditions measured by YSI sensors in the replicate aquaria over the duration of the experiment.

Temperature (C)	pH	Dissolved oxygen (mg/l)
13.0 ± 0.3	7.89 ± 0.04	9.2 ± 0.5
14.6 ± 0.2	7.70 ± 0.02	7.4 ± 0.5
15.7 ± 0.4	7.52 ± 0.07	6.0 ± 0.5

The egg mass was separated into 1–2 g cubes of egg mass and placed in each aquarium. This cube size allowed for replication across the treatments and a surface-area-to-volume ratio such that eggs at the center of the cubes experienced the treatment conditions. The cubes were taken from the periphery of the egg mass, separated by hand, quickly dried with kim wipes, and weighed. We weighed 20 individual eggs from the full mass to calculate an average weight per egg and estimate the number of eggs per 1–2 g cube. Once weighed, the egg cube was placed on top of a 10 × 10 cm ceramic tile situated in a 500 micron mesh aquarium bag, and secured with a rubber band. The tile ensured the egg cubes rested on the bottom of the aquaria and the mesh bag made certain day-1 Lingcod larvae could not escape from the aquaria.

A total of four aquarium bags were placed in each replicate aquarium; one bag was reserved for examining metabolic rates and the other three bags were left undisturbed until hatch. Placement of the four bags was rotated every other day to ensure all bags experienced similar conditions near the aquarium pump, inflow, and outflow pipe.

### Analysis of eggs and larvae

#### Does the metabolism of Lingcod embryos increase with environmental change?

We measured the oxygen consumption rate of Lingcod eggs as a proxy for standard metabolic rate using fiber optic O_2_ sensors (Fibox IV, Presens). The respirometry trials took place at the same degree-day (time in days, adjusted for temperature) development across treatments.

We ran the trials using 100 ml airtight containers fitted with an oxygen sensor spot (PreSens SP-PSt4-SA) that were initially calibrated with both 100% and 0% O_2_ saturated water. One egg cube from each replicate aquaria (n = 6) was added to the containers and placed on top of a magnetic stir plate in a temperature-controlled water bath. Water movement was maintained inside the containers using a magnetic stir bar that was separated from the eggs by a porous plastic platform. The oxygen concentration of each container was measured at seven timepoints over ~ 1.5 h and the eggs were sacrificed at the end of the trial. We used the R package LoLinR, which uses local linear regression techniques to determine the rate of oxygen depletion in μmol/min/g for each container and then corrected for the egg cube weight^79^.

#### Is Lingcod hatch timing shifted and does percent viable hatch decrease with environmental change?

Hatch began on March 1st and was complete by March 6th for all treatments. Once a day, hatched larvae were removed from each sample bag using a pipette, counted, and placed in a 15 ml falcon tube. The larvae were frozen in a 30% ethanol solution. Both calendar and degree days to first hatch and 50% hatch were determined for each bag (3 bags per aquarium, 6 aquaria per treatment) and the mean and standard deviation for the three treatments was calculated. Total number of hatch days was also calculated based on the number of days between the appearance of the first and last larvae.

Percent hatch for each bag was the number of hatched larvae counted, including deformed larvae, divided by the estimated number of eggs in the cube × 100. Percent viable hatch was also calculated using the number of larvae without deformities, viewed using a dissecting microscope.

#### Does larval quality decrease with environmental change conditions?

**Figure 6:**
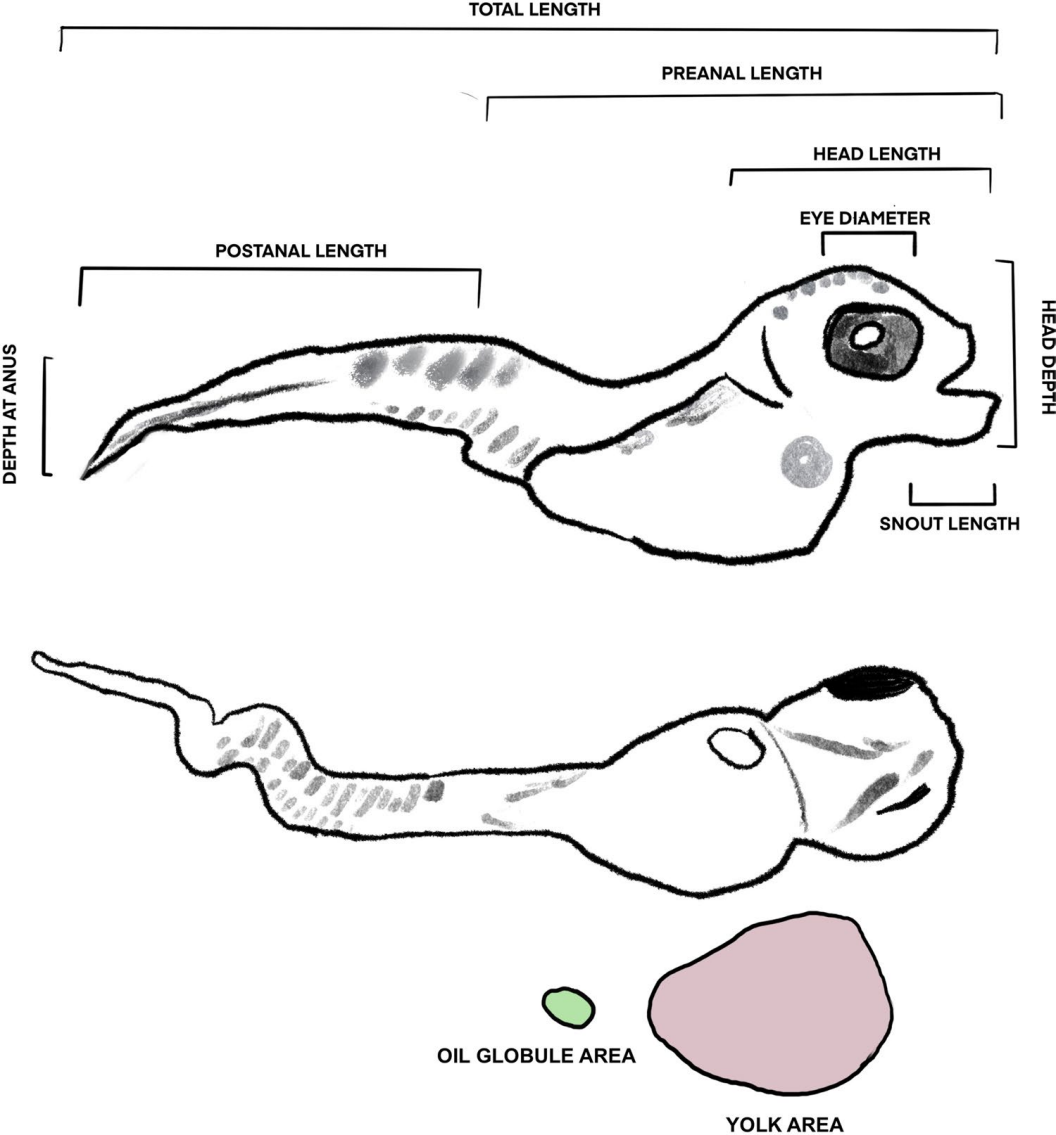
Illustration of the 10 measurements taken for each larval image using ImageJ.

To determine differences in larval quality between treatments, twelve metrics were used: (1) total length, (2) preanal length, (3) postanal length, (4) head length, (5) snout length, (6) eye diameter, (7) head depth, (8) depth at anus, (9) oil globule presence/absence and area, (10) yolk area, (11) number and type of deformity, and (12) dry weight (Fig. 6). To complete the morphometric analyses, six larvae from each falcon tube were randomly selected (a bootstrap analysis determined six larvae was a sufficient sample size) and images from the ventral and lateral side were taken using a microscope camera (AmScope 10 MP). We visually identified the type of deformity for each larvae and then the program ImageJ was used to record the measurements. To increase confidence in our measurements of each image, we used the mean of measurements by six different individuals. After images of the larvae were taken, the larvae were freeze-dried for 48 h and weighed to the nearest 2 μg on an analytical balance (Sartorius).

The amount of total carbon, total nitrogen, carbon-13, nitrogen-15, and the carbon to nitrogen ratio (C:N) was measured for each larva to compare body composition between treatments. To complete the analysis, samples were weighed, encapsulated in tin, and analyzed for C and N stable isotope ratios and C and N amounts by the University of California Santa Cruz Stable Isotope Laboratory using a CE Instruments NC2500 elemental analyzer coupled to a Thermo Scientific DELTAplus XP isotope ratio mass spectrometer via a Thermo-Scientific Conflo III. Measurements are corrected to VPDB (Vienna PeeDee Belemnite) for δ13C and AIR for δ15N against an in-house gelatin standard reference material (PUGel), which is calibrated against international standard reference materials. Measurements are corrected for size effects, blank-mixing effects, and drift effects. An externally-calibrated Acetanilide standard reference material purchased from Dr. Arndt Schimmelmann of Indiana University is measured as a sample for independent quality control. Typical reproducibility is significantly better than 0.1 permil for δ13C and significantly better than 0.2 permil for δ15N.

### Statistical analyses

Nested mixed model ANOVAs, with treatment as the fixed effect and tank as the random effect, were used to test statistical significance (p < 0.05) between treatments for the respirometry, morphometric, and stable isotope analyses, all normally distributed datasets. Generalized linear mixed models with a beta distribution were created using the R package glmmTMB to test the statistical significance between treatments for total percent hatch and percent of larvae with deformities^80^. Generalized linear mixed models with a Poisson distribution were used to test the statistical significance between treatments for days to first hatch, days to 50% hatch, and total number of hatch days.

## Supplementary Information

Supplementary Information 1.

Supplementary Information 2.

## Data Availability

Sensor data analyzed for the current study are available from the corresponding author on reasonable request. All other datasets generated and analyzed during the current study are available in the article’s Supplementary Information files.
