# Supplementary material for: Multistressor global change drivers reduce hatch and viability of Lingcod embryos, a benthic egg layer in the California Current System
Source: Sci Rep. 2022 Dec 20;12:21987. doi: 10.1038/s41598-022-25553-z (PMC9768118; doi:10.1038/s41598-022-25553-z)
Supplement: Supplementary file 2 — Supplementary Information 2. [file 41598_2022_25553_MOESM2_ESM.docx]

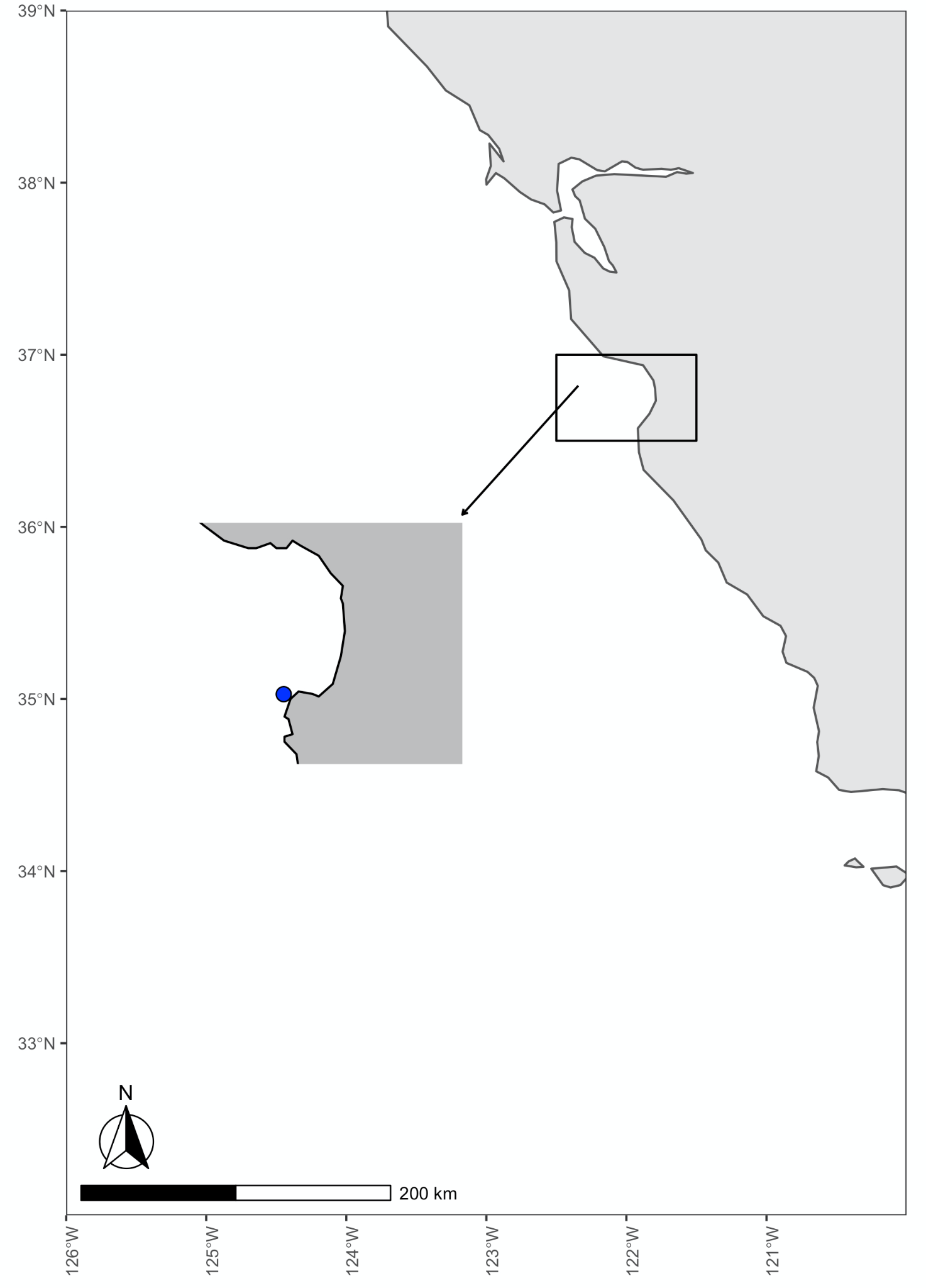


**Supplementary Figure 1:** Map of Monterey Bay, CA (black rectangle) showing location of lingcod egg mass collection (blue circle).

|  | **2020** | **2050** | **2100** |
| --- | --- | --- | --- |
| **Temperature ^o^C** | 13.09 ± 0.16 | 14.69 ± 0.21 | 15.82 ± 0.23 |
| **Salinity (ppt)** | 34.46 ± 0.05 | 34.45 ± 0.06 | 34.46 ± 0.05 |
| **Dissolved Oxygen (mgL^-1^)** | 9.57 ± 0.62 | 7.71 ± 0.50 | 6.37 ± 0.56 |
| **pH** | 7.89 ± 0.04 | 7.70 ± 0.03 | 7.53 ± 0.06 |
| **TA (μmol kg^-1^)** | 2246.9 ± 4.0 | 2246.51 ± 3.6 | 2248.81 ± 5.05 |
| **HCO_3_- (μmol kg^-1^)** | 1961.94 ± 15.21 | 2058.90 ± 9.34 | 2108.28 ± 18.39 |
| **pCO2 (μatm)** | 535.10 ± 41.94 | 968.79 ± 52.81 | 1457.18 ± 192.68 |
| **CO_2_ (μmol kg^-1^)** | 21.26 ± 1.65 | 36.47 ± 1.89 | 53.09 ± 6.83 |
| **CO_3_^2-^ (μmol kg^-1^)** | 113.78 ± 6.67 | 74.83 ± 3.33 | 55.54 ± 7.20 |
| **DIC (μmol kg^-1^)** | 2096.98 ± 10.33 | 2170.20 ± 7.20 | 2216.90 ± 18.08 |
| **ΩCalcite** | 2.72 ± 0.16 | 1.79 ± 0.08 | 1.33 ± 0.17 |
| **ΩAragonite** | 1.74 ± 0.10 | 1.15 ± 0.05 | 0.85 ± 0.11 |

**Supplementary Table 1:** Discrete water samples were collected at three time points (2/12, 2/21, 3/2) from each replicate aquaria (n=6 per treatment) during the experiment. Tris buffer-calibrated YSI sensors measured pH, temperature, DO, and salinity at the time of discrete water sample collection. Total alkalinity from these discrete samples was measured using a Metrohm 815 Robotic USB Sample Processor XL and Titrando 905. The CO2SYS package was used to obtain the detailed state of the carbonate system.


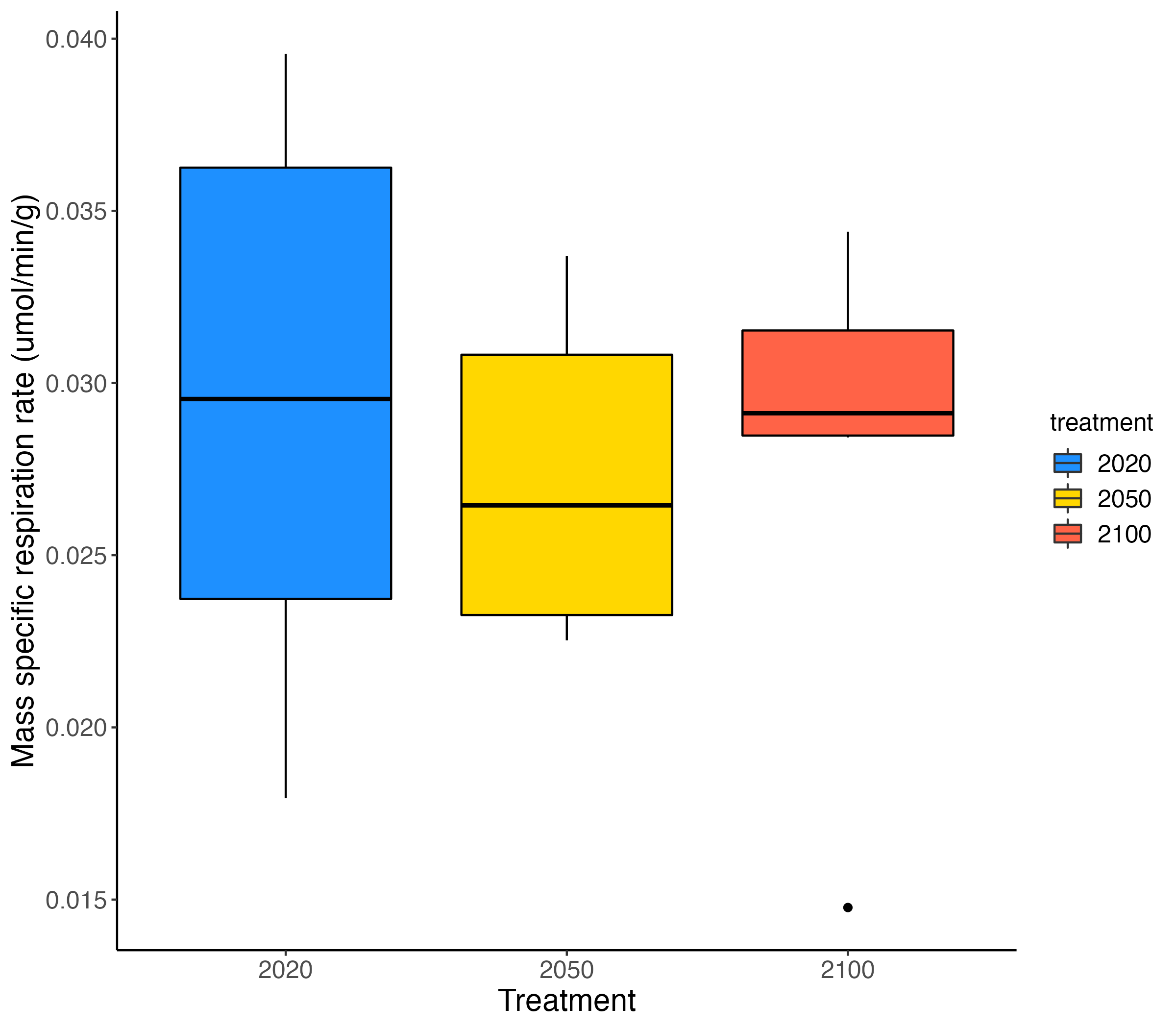


**Supplementary Figure 2:** Boxplots illustrating the median, upper and lower quartile, and interquartile range of mass specific respiration rates (μmol/min/g) for Lingcod embryos placed in the year 2020 (blue), year 2050 (yellow), and year 2100 (red) treatments. P>0.01 for all pairwise comparisons (p_2020,2050_=0.85, p_2050,2100_=0.98, p_2020,2100_=0.93)


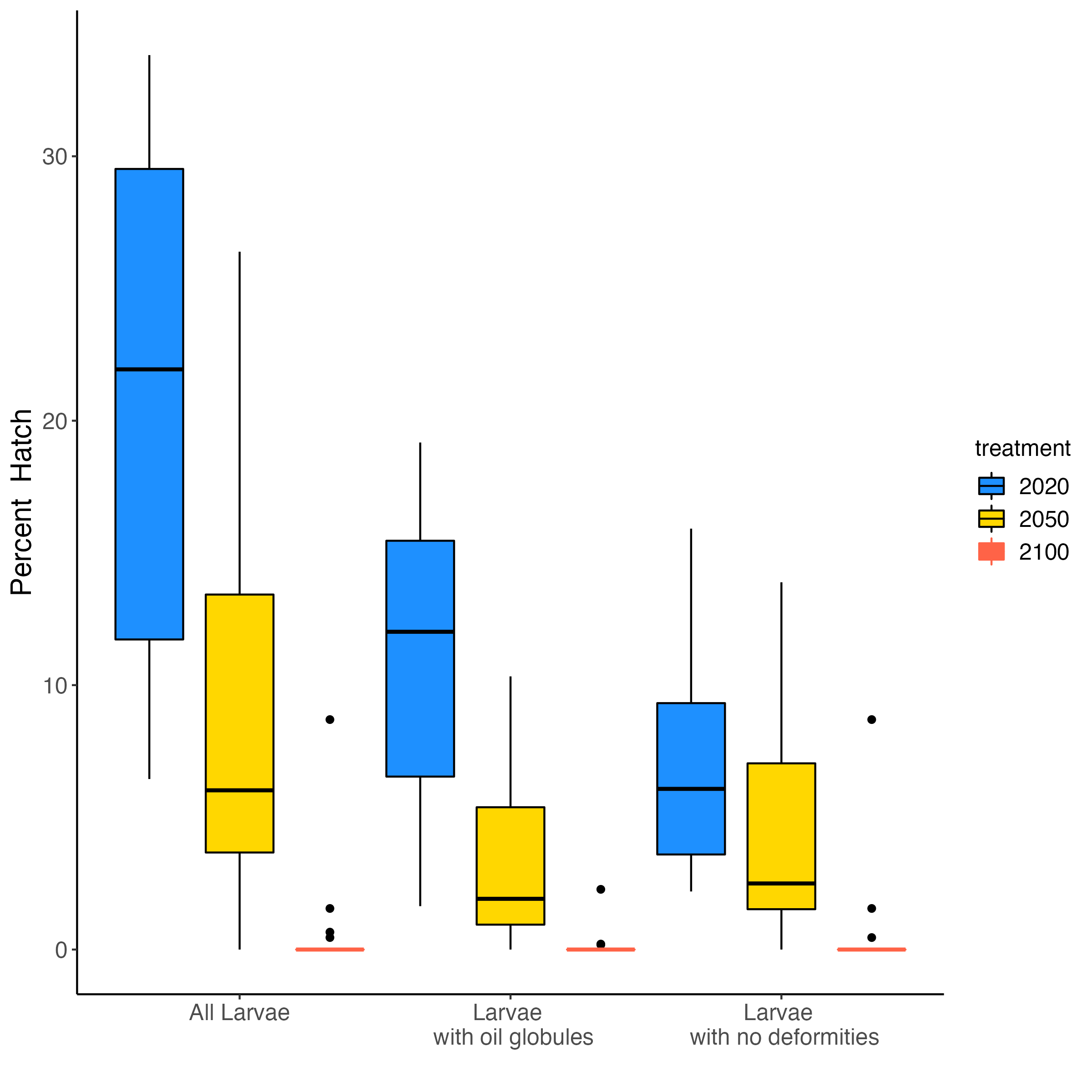


**Supplementary Figure 3:** Boxplots illustrating the median, upper and lower quartile, and interquartile range of percent total hatch, percent hatch of larvae with oil globules, and percent hatch of larvae without deformities for the year 2020 (blue), year 2050 (yellow), and year 2100 (red) treatments. Nested mixed model ANOVAs were run to determine statistical significance. The percent of larvae with oil globules was significantly lower in the future condition treatments compared to the year 2020 treatment (p_2020,2050_<0.001, p_2050,2100_=0.06, p_2020,2100_<0.001). The percent of larvae with deformities was statistically similar between the year 2020 and year 2050 treatment; the percent of larvae with deformities significantly increased in year 2100 conditions (p_2020,2050_=0.1, p_2050,2100_=0.01, p_2020,2100_<1x10^-4^).


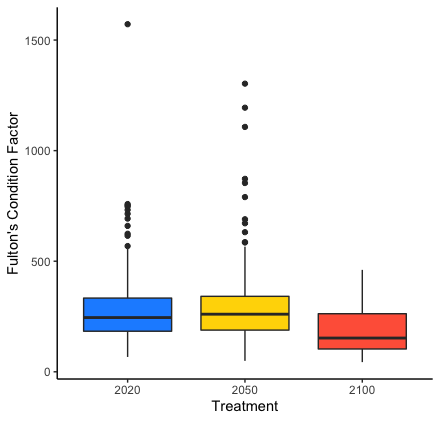


**Supplementary Figure 4:** Boxplots illustrating the median, upper and lower quartile, and interquartile range of Fulton’s Condition Factor for the year 2020 (blue), year 2050 (yellow), and year 2100 (red) treatments. There was no significant difference in Fulton’s Condition Factor between treatments.
